# Hand-based multibiometric systems: state-of-the-art and future challenges

**DOI:** 10.7717/peerj-cs.707

**Published:** 2021-10-07

**Authors:** Anum Aftab, Farrukh Aslam Khan, Muhammad Khurram Khan, Haider Abbas, Waseem Iqbal, Farhan Riaz

**Affiliations:** 1 National University of Sciences and Technology, Islamabad, Pakistan; 2 Center of Excellence in Information Assurance (CoEIA), King Saud University, Riyadh, Saudi Arabia

**Keywords:** Multimodal biometrics, Fusion techniques, Multibiometric template protection, AI

## Abstract

The traditional methods used for the identification of individuals such as personal identification numbers (PINs), identification tags, etc., are vulnerable as they are easily compromised by the hackers. In this paper, we aim to focus on the existing multibiometric systems that use hand based modalities for the identification of individuals. We cover the existing multibiometric systems in the context of various feature extraction schemes, along with an analysis of their performance using one of the performance measures used for biometric systems. Later, we cover the literature on template protection including various cancelable biometrics and biometric cryptosystems and provide a brief comment about the methods used for multibiometric template protection. Finally, we discuss various open issues and challenges faced by researchers and propose some future directions that can enhance the security of multibiometric templates.

## Introduction

Biometric authentication is used more than ever for authentication of individuals in a wide range of security applications. The reliance of systems on physiological attributes of the users has lately offered more simplicity and reliability at the same time. This has helped in avoiding many problems associated with the systems where passwords/credentials are being used, which can potentially incur some problems such as forgotten passwords, transferred or stolen credentials. The use of biometrics has led to mitigate these problems significantly given that the individual with specific biometric traits is required to validate access to the systems to avoid the above mentioned problems. Moreover, most of the existing systems are typically connected to networks, at the very least, a local area network connecting a local network with a couple of systems and more often, a wide area network eventually connecting to the World Wide Web. Given this, a protection mechanism is required to be in place to ensure that an unauthorized access to the system is prevented and the templates are properly protected.

There is a wide use of authentication systems in Internet services and mobile devices for the protection of the user content. Various tools and techniques for the management of information security have been developed. However, systems based on biometrics have made significant progress to support some aspects of information security over the period of time. An in-depth and comprehensive study on biometric authentication has been conducted in recent years by various researchers (Jain, Ross & Prabhakar, 2004; Jain, Nandakumar & Ross, 2016). With the passage of time, biometric authentication of the users is gaining more and more popularity since the systems based on biometrics are not easily compromised. This is because, the systems can be breached only if the individuals who are trying to access the systems are in possession of those physiological parameters, which are possessed by the actual users. This has led to the addition of security for the protection of the systems, and reduced their vulnerability.

It goes without saying that the field of biometrics is very rich and up to the minute. There are a number of surveys that exist on biometric systems. However, some surveys focus mainly on a particular modality or environment; some of the recent contributions include Connor & Ross (2018), in which the authors focused on a biometric recognition system based on gait. They have reviewed several gait recognition modalities and their features. Kumari & Seeja (2019) provides an in depth survey about periocular biometrics, using various existing feature extraction methods and matching schemes. The paper also emphasizes the importance of periocular biometrics in a wide range of applications. Dargan & Kumar (2020) have done a very comprehensive and in depth survey on various unimodal and multimodal biometric recognition systems discussing feature extraction methods, various classifiers and datasets. Their main aim is to make the researcher aware of multiple dimensions to look for in a biometric system in order to enhance its security. Sundararajan & Woodard (2018) have performed a survey on the use of deep learning in the domain of biometric authentication using various modalities. However, their conclusion is that most of the deep learning approaches have been explored mainly on face biometrics and speaker recognition. Dinca & Hancke (2017) emphasized the importance of multibiometric systems in their work for meeting the emerging security demand in the field of authentication. Their work is mainly focused on covering two important aspects in biometric systems: fusion methods and security. A thorough review of secure and privacy preserving authentication is presented in Rui & Yan (2018). The authors have mainly tackled the problem of liveness detection and privacy protection in biometric systems. Given an ever increasing work done in the area of wearable technology and IoTs, the wearable biometrics is another upcoming area that requires a significant attention of the researchers. In this context, Sundararajan, Sarwat & Pons (2019) have performed an interesting review in which the authors compare the key characteristics of different modalities, and highlight critical attacks being carried out in traditional and wearable biometric systems. However, the scope of the manuscript is quite broad with a review covering most of the biometric modalities including behavioral and physiological traits. Moreover, there is a lack of presentation of the quantitative analysis in several manuscripts creating a gap for a more focused and thorough review on hand based multibiometric systems.

### Rationale for the survey

There is a wide range of biometric traits that can be used for authentication purposes including face, hands, iris, retina, etc. All these traits offer their own advantages and drawbacks and most of them are thoroughly covered in the literature. In this paper, we aim to focus on the existing hand based multibiometric systems explored over the past five years. The use of hand modalities offers several advantages over others: they are highly accurate for recognition, generally make use of inexpensive technology, are fast for matching and require templates of very small sizes, resulting in a small memory footprint and are less sensitive to imaging conditions. Moreover, the hand-based modalities are more robust since they are not affected by emotions and other behavioral characteristics of the individuals such as tiredness, stress, etc. Given this, it is clear that with respect to some specific aspects, the use of hand based modalities is superior as compared to others for biometric authentication.

There is a large volume of literature discussing about the use of hand based modalities for authentication but the work becomes limited as it is directed towards multimodal systems. This domain was constrained until recently due to the issues related to the power consumption, size, and cost, etc., of the hardware required for executing the biometric systems. However, over the recent years, the revolution in the hardware design industry has led to miniaturized devices having tremendous capabilities, enabling the developers to execute multiple systems on very light, low-powered, and small devices with significantly lesser cost. Consequently, it has been possible to implement multibiometric systems very efficiently, triggering a lot of research in this direction. Moreover, to the best of our knowledge, a thorough survey of hand based multibiometric systems, their effective usage in biometric authentication and the main challenges faced, is currently not available in the literature.

With regard to the security of the biometric system, being multibiometric in nature adds itself another layer of security even though there are multiple points of attack on an authentication system. We aim to tackle the literature available regarding security of multibiometric templates. The rationale for focusing on security of multibiometric template is that they lead to a 3-dimensional vulnerability to a biometric system in contrast to their counterparts (Jain, Nandakumar & Nagar, 2008): 1. Template can be replaced by an imposter to gain unauthorized access, 2. A spoof can be created from the template to aide in unauthorized access, and 3. The stolen template can be replayed to the matcher to gain access. Therefore, it is vital to protect the templates from an adversary; unlike PINs and passwords, a biometric template if compromised cannot be revoked and reissued, so considering the criticality in this context, we aim to deal with the research contributions that are devised to protect the integrity of the saved templates.

This paper presents a systematic review on the use of hand based multibiometric systems and an analysis of their efficacy in performing authentication. In this context, the main contributions are as follows:

- Discussing the main advantages and motivations behind the usage of hand based multibiometric systems.
- Presentation of a taxonomy to categorize the literature with respect to the two parameters: authentication and template protection.
- Presentation of a summary of literature on the above-mentioned parameters, with critical/brief comments.
- A discussion about open issues and the direction of future work on hand based multibiometric systems.

In summary, this paper covers two major directions of work on hand based multibiometric systems: 1. The work on various schemes to perform feature extraction and authentication of individuals using multibiometric systems based on hand modalities. The main objective of such studies is to ensure that the performance metrics of the systems are very good and they can be effectively used, and 2. Once the templates have been acquired from the users (the users are enrolled), how these templates can be effectively archived such that they are not susceptible to attacks.

### Paper organization

The rest of the paper is organized as follows: In “Survey Methodology”, we present the methodology followed in this survey. We discuss an overview of a biometric system, multibiometric system and its types in “Overview of a Biometric System”, followed by the discussion on fusion methods used in a multibiometric system in “Fusion Methods”. Later, we talk about different hand-based modalities that are used for biometric authentication in “Hand Based Modalities”. Then, we discuss the feature extraction methods in existing hand multibiometric systems in “Feature Extraction Techniques for Hand Multibiometric Systems” and methods used to perform multibiometric template security in “Multibiometric Template Security”. Finally, we discuss about various open issues and challenges in the topics covered in “Open Challenges and Future Directions” and conclude the paper in “Conclusions”.

## Survey methodology

With an ever growing demand of designing authentication systems and the linkages of such systems with critical databases owned by the governments, corporates and various entities, there is an increasing demand on making these systems scalable, user interactive, safe, and secure. In this context, the biometric technology has significantly grown over the last decade. Subsequently, several works have been conducted listing the major contributions and breakthroughs in the area. However, to the best of our knowledge, there is a shortage of detailed surveys on the use of hand-based modalities for multibiometric systems and an analysis of security aspects with respect to template protection. The recent contribution on this topic was done by Bahmed & Mammar (2019). However, the survey is limited and lacks a thorough analysis and discussion on future directions and does not cover the security aspect of the biometric systems. Moreover, there is a need to define a clear taxonomy that helps in defining the future research directions in the subject area.

**Figure 1:**
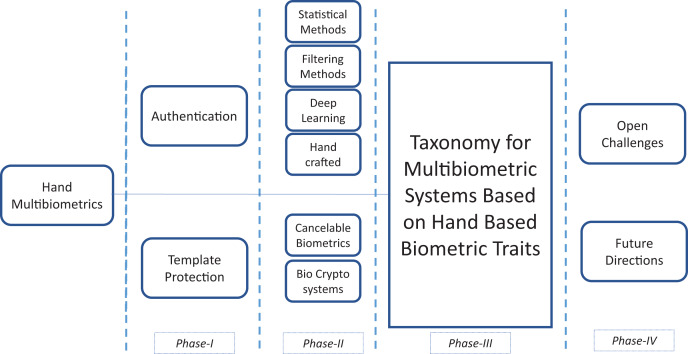
Survey methodology.

In this survey, the approach followed to collect the manuscripts is shown in Fig. 1. The scholarly databases used for searching articles were IEEE explore, ACM Digital Library, Springer, Science Direct and Google Scholar. Most of the identified research papers are published in reputed forums. We have mainly focused on papers published from 2010 till date (May 2021). The search terms used for collecting the manuscripts were: “Multibiometric systems” with permutations of different hand modalities. According to the search terms, more than 600 papers were initially identified which were screened to fit the scope of the topic based on their title, abstracts, body, and conclusions. For our work on authentication using hand based multibiometric systems, we have identified 35 manuscripts, whereas for multibiometric template protection, we have picked out 22 manuscripts. We have followed a semi-systematic methodology for this survey (Snyder, 2019), narrowing down the literature in multiple phases. Breadth search is first adopted in which the literature on all hand multibiometric systems is analyzed. It was concluded that the optimization parameters on multibiometric systems revolved around two important performance primitives: authentication and security. These are complementary parameters as optimizing security may compromise on the authentication results obtained for the systems, although, a vice versa may not be necessarily true. Therefore, this led us to the Phase II of our research in which we conduct a depth search to shortlist the literature based on the design of authentication of multibiometric systems and template security. Phase II is formalized as a taxonomy in Phase III, which structures the literature for a better understanding and comprehension of the underlying problem. The inference drawn from an evaluation of the literature has led to a discussion about some challenges leading to subsequent future directions of work in this domain (Phase IV).

## Overview of a biometric system

In a biometric system, an identifier is linked to its intrinsic human characteristics. These characteristics are physiological and behavioral in nature, which can be used to identify a person digitally (Meng et al., 2014; Rui & Yan, 2018). Biometric security helps in authentication, which takes place by identifying human characteristics. The specific human characteristics mentioned above are defined as follows:

- Physiological: Physiological biometrics are based on physical characteristics of an individual. They vary from individual to individual and are assumed to be relatively unchanging such as fingerprints, face, iris/retina etc.
- Behavioral: Behavioral biometrics are based on behavioral characteristics of an individual. The examples include voice, gait, signature, etc.

**Figure 2:**
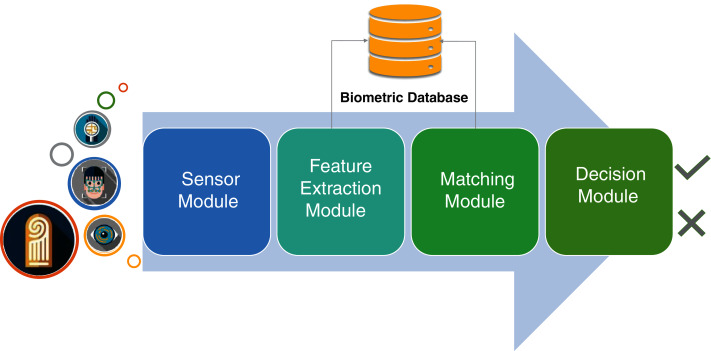
Logical blocks of a generic biometric authentication system.

There are four important modules in a traditional biometric system (Fig. 2). The sensor module is responsible to acquire data from the users, whereas the feature extraction module processes the sensor data to find a description that is feasible for matching of templates that are residing in the database. The feature extraction module is followed by the matching module that generates the matching scores, which are finally used to perform the decision making regarding the grant of permissions to a specific user in the decision module.

The several factors that are considered significant while performing the selection of a specific biometric identifier include permanence, universality, measurability, circumvention, performance, etc (Bhattacharyya et al., 2009). Another important factor is the suitability of the application. Nevertheless, the choice of a single biometric identifier which meets all the requirements of every possible application is not possible since there are tradeoffs between different performance metrics. Keeping this in view, there is a possibility to optimize a number of measures by using a combination of various biometric identifiers. Therefore, we can logically characterise a biometric system into two distinct categories: (1) Unibiometric systems, and (2) Multibiometric systems.

### Unibiometric systems

Traditionally, biometric recognition systems are unibiometric, which employ a single biometric trait for authentication purposes. It may use one of the physical or behavioral biometric traits, such as fingerprint recognition (Maltoni et al., 2009; Hong, Wan & Jain, 1998; Yuan, Sun & Lv, 2016), face recognition (Zhao et al., 2003; Masi et al., 2018; Wang et al., 2018), iris recognition (Nguyen et al., 2017), signature, etc. In the literature, the use of unibiometric systems is widely employed with very good recognition results. However, such systems are typically constrained due to several factors including lack of accuracy due to noisy data, non-universality of biometric traits for registration, physiological limitations of biometrics, and vulnerabilities in biometric systems (Table 1) (Dinca & Hancke, 2017; Oloyede & Hancke, 2016).

**Table 1:** Issues associated with unibiometric systems.

Name	Description
Noise in data	The acquisition environment corrupts the data due to which the features are altered. This can result in a false registration of the user.
Lack of universality	A certain biometric trait cannot be used due to some clinical condition such as a cut in the finger, long eyelashes resulting in an iris failure, etc.
Identification accuracy	For large databases, a certain biometric trait will be able to handle a maximum number of distinguishable patterns after which it will not be able to discriminate between the users.
Spoofing	Some behavioral and physical biometric traits are vulnerable to attacks, *e.g*., signatures and even fingerprints. Successful presentation of a spoofed biometric will result in an authentication compromise.

Some biometric modalities are more vulnerable to some specific problems, *e.g*., spoofing a fingerprint is relatively easier as compared to a vein/palm pattern. However, the recognition accuracy of fingerprints is far more superior. These are complementary properties of two different biometric modalities, which can be exploited together in a multimodal biometric system, hence making the system more tolerant to spoofing while maintaining a higher accuracy.

### Multibiometric systems

**Figure 3:**
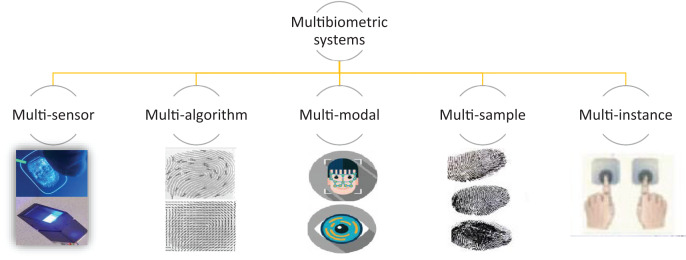
Types of multibiometric system.

When using the unibiometric systems, we may encounter problems due to several issues including, but not limited to, missing data (Nandakumar, Jain & Ross, 2009) (*e.g*., occlusion in face image), poor sampling (Grother & Tabassi, 2007), biometric duplication (Sudhish, Jain & Cao, 2016), low discrimination among samples (*e.g*., hand shape/geometry) between distinct users, vulnerability to attacks, and spoofing, etc (Jain & Kant, 2015). In situations like these, it may be necessary to make use of multiple biometric cues to boost the accuracy of a recognition system. The multibiometric systems offer so many features, making them more convenient and feasible as compared to the unimodal systems. There can be different sources of biometric information in a multibiometric system due to which such systems can be classified into the following five major categories (Fig. 3):

#### Multi-sensor systems

The multi-sensor systems use multiple sensors in order to capture the same biometric trait of an individual (Elhoseny et al., 2017; Kaur & Sohal, 2017). Such systems are desirable due to the fact that they can enhance the recognition capabilities of the systems (Blum & Liu, 2005). This happens because the data acquired from various sensors may be of different quality and the multi-sensor system can partially solve the problems related to poor data quality (Singh, Singh & Ross, 2019). The different biometric traits, when used for recognition, have the ability to complement one another, creating the possibility for a better recognition of the individuals.

#### Multi-algorithm systems

In multi-algorithm systems, more than one algorithm are utilized to improve the recognition rates of biometric systems (Sotonwa & Oyeniran, 2019; Gad et al., 2018). It is cost effective to work on such systems as they do not make use of multiple biometric traits, and thus do not require multiple sensors (Mishra, 2010). However, such systems require a lot of computational resources as multiple algorithms have to be run in order to calculate the relevant features for a single instance (Fan et al., 2019; Scherhag, Rathgeb & Busch, 2018). Keeping this in view, special consideration should be given to the fact that real-time performance is a requirement of biometric systems, and thus the feasibility of such systems might be compromised even when they have the ability to achieve very high recognition rates.

#### Multi-sample systems

In multi-sample systems, multiple samples from the same sensor are acquired from the biometric devices (Dinca & Hancke, 2017; Elhoseny et al., 2018). The fundamental issue with a single sample system is the fact that the samples can suffer from missing data problems due to which effective recognition cannot be performed (Goswami et al., 2017). The problem is mitigated in multi-sample systems by acquiring multiple samples from the devices and using multiple or the most relevant samples for recognition. The same algorithm is used to process all samples and recognition results from each sample are calculated and eventually fused to yield a final result of recognition (Modak & Jha, 2019). This recognition may be based on some technical considerations, *e.g*., a confidence score with which a specific recognition result is obtained.

#### Multi-instance systems

In multi-instance systems, the biometric data is typically extracted from multiple instance of the same body traits (Faltemier, Bowyer & Flynn, 2008). For example, finger biometric properties can be extracted from two fingers (Lamia & Najoua, 2019), the palm prints can be acquired from two palms (Leng et al., 2017), and the iris of the individuals (Kumar, Prasad & Raju, 2020) can be used for measuring different biometric traits of the systems. The addition of multiple instances for performing recognition in a biometric system increases the discrimination capability of the system because the distinctive capability for a single individual is extended by adding more features to the pool, potentially leading to an improvement in the recognition rates for a system (Leng et al., 2017).

#### Multi-modal systems

In the multi-modal systems, the biometric traits from different modalities can be combined together for the purpose of identification of an individual (Modak & Jha, 2019). Such systems are used to complement the weaknesses of a single biometric and they usually try to make the best of different biometric traits in order to perform recognition of an individual (Yang et al., 2018b). An additional advantage of using multi-modal biometric systems is that they are more secure as compared to the uni-biometric systems as more than one trait is used at the time of registration of a user in a system (Yang et al., 2018b; Barni et al., 2019; Gomez-Barrero et al., 2017). Appropriately, stealing or forging one biometric trait does not guarantee an access to the system, thus leading to an improved security feature for authentication in biometric systems.

Designing a multibiometric system has a very high significance. A valid design will be able to ensure that the pieces of evidence collected from various sources, when fused together using different fusion strategies, can improve the recognition rates while ensuring some value added services provided to the users. However, when different modalities have to be combined to implement multibiometric systems, special consideration has to be given to several dimensions, *e.g*., what kind of additional sensors will be required, what are the costs, is there a possibility to embed different sensors in the device and, what is the overhead of such a system in terms of computational complexity.

### Performance metrics for evaluation

Multiple metrics can be employed to assess the performance of a biometric authentication system. Choosing a particular metric(s) depends upon the nature of evaluation. Following are the basic raw metrics and their descriptions:

- **True Accept (TA)**: A genuine user is correctly verified to its corresponding template stored within the biometric system.
- **True Reject (TR**): An imposter is correctly rejected as its data does not match any template stored within the biometric system.
- **False Accept (FA)**: An imposter is incorrectly verified as a genuine user as his data is matched to the template stored within the biometric system.
- **False Reject (FR)**: A genuine user is incorrectly rejected as his data does not match any template stored within the biometric system.

The standard metrics that have been used to evaluate the performance of the authentication system in the literature are as follows:

- **False Accept Rate (FAR)**: Describes the percentage of impostors that were incorrectly verified as genuine users. It is calculated on the basis of the following formula:
(1) $$FAR = \displaystyle{{FA} \over {FA + TR}}$$
- **False Reject Rate (FRR)**: Describes the percentage of genuine users that were mistakenly rejected from a biometric system. It is calculated on the basis of the following formula:
(2) $$FRR = \displaystyle{{FR} \over {TA + FR}}$$
- **Correct Recognition Rate (CRR)**: It gives the probability that the system will correctly identify the input template from the templates in the database. It is given by the formula:
(3) $$CRR = \displaystyle{{TA} \over {TA + TR}}$$
- **Genuine Acceptance Rate (GAR)**: Describes the percentage of genuine users accepted by the biometric system. It is given by the formula:
(4) $$GAR = 100 - FRR$$
- **Accuracy**: It is the ratio between verified cases (both True Accept and False Accept) to all possible cases. It is given by the formula:
(5) $$Accuracy = \displaystyle{{TA + FA} \over {TA + FA + TR + FR}}$$
- **Equal Error Rate (EER)**: Describes the point at which FAR and FRR are equal. Smaller values of EER refers to improved performance of a biometric system.

## Fusion methods

**Figure 4:**
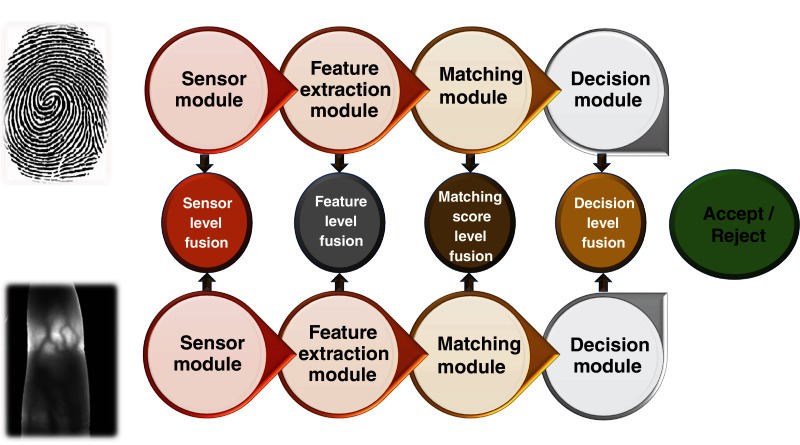
Fusion levels in a multibiometric system.

Fusion plays a very considerable role in the implementation of multibiometric systems. There is an inherent requirement to fuse the information collected from different modalities before using it for the purpose of recognition. Fusion can be applied in multibiometric systems in two major settings: before matching and after matching. Consequently, there are five distinct levels at which fusion can be applied, *i.e*., sensor level, score level, feature level, and decision level (Fig. 4). The fusion applied at the first two levels is referred to as pre-matching fusion, whereas the rest are categorised as post-matching fusion.

### Sensor level fusion

In sensor level fusion, the raw information is gathered from various sensors and is fused at the initial level prior to feature extraction to produce a raw fused information. Fusion of two images can take place at pixel, feature or at signal level. Fusion at sensor level can be between multiple samples of the same biometric gathered from multiple sensors (Yang et al., 2005) or multiple instances of the same biometric taken from a single sensor (Othman & Ross, 2012). Relatively less research has been done on this type of fusion in biometrics.

### Feature level fusion

In feature level fusion, the features extracted from multiple biometric sources are combined together in the form of a single feature vector. In this fusion technique, features from different sensors, samples, and traits can be combined together. At this level of fusion, signals from various biometric channels are firstly pre-processed and their feature vectors are calculated independently; by using fusion algorithms, the feature vectors are fused to form a combined feature vector, which is used for recognition (Xin et al., 2018). The incorporation of multimodal biometric traits in this type of fusion can be employed to exploit specific strengths of different biometric modalities (Nagar, Nandakumar & Jain, 2011; Jagadiswary & Saraswady, 2016; Prasanalakshmi, Kannammal & Sridevi, 2011). Although better recognition results can be expected using this type of fusion technique, it has certain limitations including the lack of compatibility of different biometric features, curse of dimensionality, etc.

### Matching score level fusion

This type of fusion is done by joining the scores yielded by the matching module of each feature vector with the template. The features are processed independently along with the calculation of scores, followed by the calculation of composite matching scores (He et al., 2010; Yilmaz & Yankoglu, 2016; Kabir, Ahmad & Swamy, 2018). This is done by the checking the confidence scores, which are obtained using each feature vector. This type of fusion technique is typically easy and thus is being used by different multibiometric systems for effective execution.

### Rank level fusion

In this type of fusion, sensor data is acquired followed by the feature extraction. The matching of this feature vector is performed against all the available templates in the database and similarity scores are obtained (Kumar & Shekhar, 2010; Monwar & Gavrilova, 2009). The scores are arranged in the descending order and the entry corresponding to the lowest rank (indicating similarity of feature vector with the respective template) is taken as the most relevant to the data that is acquired from the sensor. The rank level fusion can also be employed for multibiometric traits and thus can yield a recognition score with a higher confidence. However, it should be noted that in addition to the pre-processing of sensor data, additional computational load is transfered on the matching module. Therefore, the rank level fusion can be computationally very complex especially when more than one biometric trait is employed.

### Decision level fusion

In this type of fusion, the information obtained from different decision modules is combined together to decide about the identity of a user (Jiang et al., 2014; Niu et al., 2008). The recognition results of each biometric trait are individually obtained followed by a fusion of these decisions to obtain a final decision regarding recognition (Li et al., 2018; Ghosh, Sharma & Joshi, 2014). Various methods to perform this type of fusion can be used, *e.g*., majority voting can be employed (Jimenez, Morales-Morell & Creus, 1999). In systems which require enhanced security and fail safe functioning, rule based decision can also be made such as the use of a logical ‘AND’ operation, indicating that it is necessary for all biometric traits to be yielding the same output.

## Hand based modalities

As discussed previously, there are many modalities that can be used to obtain biometric information from the users. Making the right choice for designing biometric systems is a question that requires consideration in multiple dimensions. The ease of use, budget, overall performance in terms of recognition ability and modalities that promote anti-spoofing are important influential factors that determine the best biometric trait for biometric security research. A brief overview of most of the physiological biometric modalities is summarized in Table 2. The choice of the modality presents a trade-off between different factors, which require a careful review based on the nature of intended applications. As can be seen in Table 2, retina and iris present the technologies which show very good recognition results. Physiologically, these modalities are highly distinctive for different individuals with almost no chance of repetition of the patterns. However, they are not user friendly, are expensive with respect to technology and highly sensitive to the protocols used for acquisition of the data. These specific limitations have resulted in a highly restrictive use of these two modalities, specially from the perspective of the convenience of the end user. Ear is one of the most stable biometric; however, it is not distinctive and is also sensitive to some external factors such as wearing of cap, jewelry, etc. Face is a physiologically motivated biometric and is very useful; however, the most fundamental flaw with the face biometric is that it is a source of infringement of the user’s privacy. Therefore, the users are typically not comfortable with hosting of their facial data specially by the third parties.

**Table 2:** A review of pros and cons of different physiological biometric modalities.

Modality	Advantages	Disadvantages
Retina	• Retinal pattern cannot be forged	• Not user friendly
	• Highly distinctive	• Sensitive to medical conditions
	• Provides a high security in authentication	• Expensive technology
		• Requires controlled environment
Iris	• Highly accurate	• Not user friendly
	• Highly scalable	• Expensive technology
	• Iris pattern remains stable over a long time	• Requires controlled environment
	• Small template size, fast matching	• Occlusion due to eyelashes, lenses
		• Illumination should be controlled
		• Sensitive to medical conditions
Ear	• Fixed shape and appearance	• Sensitive to earrings, hats etc.
	• Most stable	• Comparatively less distinctive
Face	• Physiologically motivated: humans identify each other based on faces	• Facial traits change over time
	• Requires a standard camera	• Dependent on lightning conditions
	• Fast matching based on facial features	• Causes infringement of privacy
Fingerprint	• Inexpensive technology	• Cuts, scars etc can alter fingerprints
	• Secure and highly reliable	• Easily deceived through wax finger
	• Fast matching as template size is small	• Some people have damaged fingerprints
		• Unhygienic: physical contact with the sensor
Palm print	• Highly distinctive	• Sensitive to illumination variations
	• More reliable, permanent	• Large recognition area
	• Good results with low resolution cameras	• Scanners are bulkier and expensive
Hand vein	• Contactless and hygienic	• Age related deformations
	• Very accurate	• Relatively expensive technology
	• Difficult to forge	• Sensitive to environment
Hand geometry	• User friendly, contactless and hygienic	• Not very distinctive
	• Results not effected by external factors	• Large recognition area
		• Large storage requirement
		• Can be used only for adults
Finger knuckle	• Contactless and user friendly	• Non-uniform reflections
	• Works with low resolution	• Sensitive to environment conditions
	• Low cost	• Shortage of public databases

**Figure 5:**
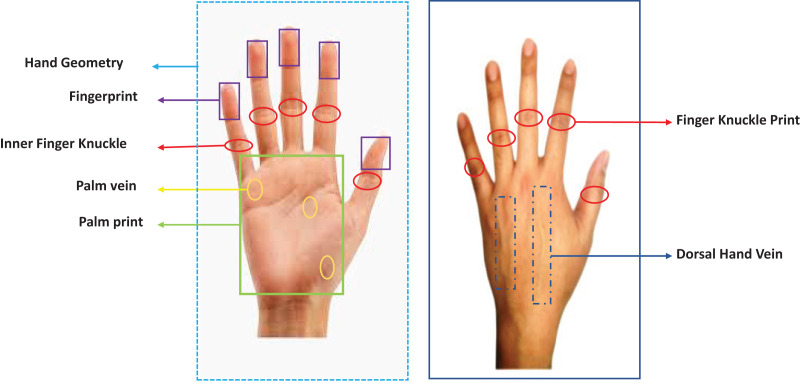
Front and back view of hands along with various biometric traits.

In contrast to the above-mentioned modalities, the remaining four, *i.e*., fingerprint, palm print, hand vein, and hand geometry are the modalities which are based on hands. It should be noted that all these modalities (except hand geometry) are highly accurate for recognition, make use of inexpensive technology in general, are fast for matching as they require templates of very small size, and are less sensitive to acquisition conditions as compared to the other modalities that are used for biometrics. Therefore, in this paper, we have focused on a thorough review of the available literature on the use of biometrics based on hands (Fig. 5).

### Fingerprint

**Figure 6:**
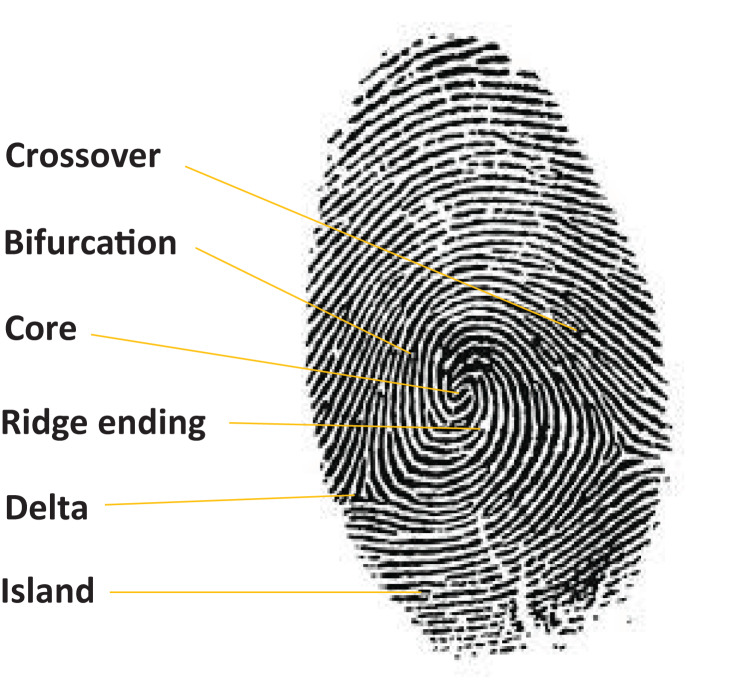
Example of a human fingerprint.

Fingerprint is one of the most established biometric modalities due to its high recognition rates and consistency, and has been in existence for over a century. The ease to acquire fingerprints and their wide usage has led to many commercial applications relying on them as far as biometrics are concerned. A fingerprint is formed by the coexistence of a collection of ridges and valleys, thus yielding a pattern, which is distinct for different human beings. These patterns are also referred to as “minutiae” and are mainly composed of bifurcations, enclosures, ridge endings and ridge dots. Further, the minutiae are subdivided into sub minutiae such as pores, crossovers, and deltas (Fig. 6). A fingerprint biometric system has four main stages: acquisition of data, feature extraction, template creation, and matching. The ease of use and a small space required for the storage of template has made it one of the best biometric technologies to employ commercially.

On fingerprint biometric, both quantitative and qualitative works exist. A survey of around 160 users was done in Arteaga-Falconi, Al Osman & El Saddik (2015) and Cappelli et al. (2007) in which the users gave a positive response towards using this technology for smartphones. Furthermore, various technological contributions presenting quantitative results show that a fingerprint take less than one second for matching, achieve 0.07% EER on a database of 100 subjects, false rejection rates of upto 0.04% and false acceptance rates of upto 0%, 4.18% and 8.91% using three confidence coefficients, *i.e*., 99.0%, 99.5% and 99.9% respectively. These results indicate a very high recognition performance in a very small amount of time, promoting the use of fingerprint technology for real time implementation of systems requiring biometric validation.

### Palm print

**Figure 7:**
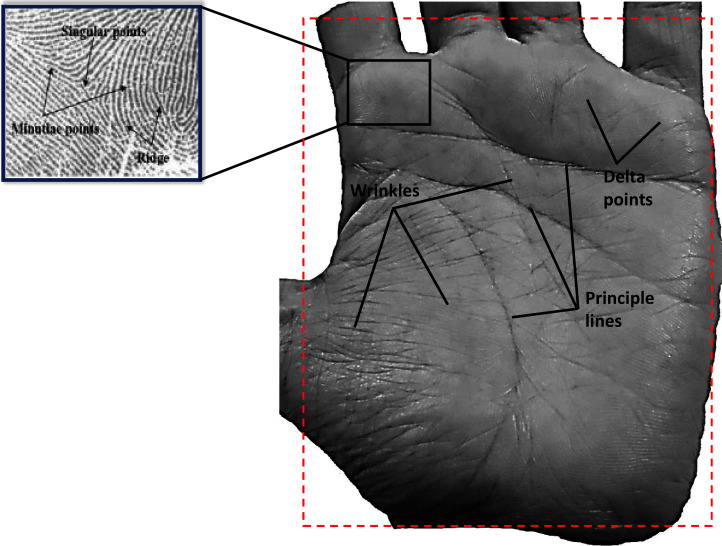
Example of human palm print.

Palm print is a popular biometric modality, which has attracted the attention of many researchers. However, it is a relatively new biometric modality as compared to its counterparts, such as face, fingerprints, etc. A palm print image consists of some rich intrinsic features such as ridges and palm lines, delta lines, principle lines, minutiae features, wrinkles, etc. (Chen, Huang & Zhou, 2013; Huang, Jia & Zhang, 2008) that are deemed to be permanent and unique for every individual (Fig. 7). Owing to these inherent features, palm prints generate unique biometric characteristics for every individual that are reliable for identification purposes (Zhang, Zuo & Yue, 2012; Zhong, Du & Zhong, 2019). The main issue that is responsible for reducing the performance of palm print systems is the deformation of images during the image acquisition process. Attempts are being made to solve this issue by using contact devices, but researchers have faced several challenges in the design of such devices including its size and limited usability, along with several challenges including position, stretch and rotation of the palm print. Lately, researchers are resorting to contactless devices again with low resolution imagery used for commercial application and high resolution imagery for applications such as criminal investigation.

Some of the most recent contributions on palm print biometric (Zhang et al., 2017; Tabejamaat & Mousavi, 2017) show that the recognition rates of upto 98.78% and 97.2% respectively have been achieved within processing times in the order of milliseconds on databases of fairly large size (about 12,000 instances). The most relevant contribution in this regard with the best accuracies are presented by Luo et al. (2016) in which the authors have reported an accuracy of 100% on a dataset having 4,600 instances of palm prints. This is in contrast to a general perception that palm prints are not as accurate as fingerprints. However, it should be noted that the possibility to obtain a relatively large dataset to validate the findings on fingerprints having a statistically higher significance is much more likely in contrast to palm prints for which the availability of dataset is relatively limited.

### Veins

Vein biometrics, also known as vascular biometrics, refer to a biometric system that measures parts of an individual’s circulatory system for identification. Vein pattern recognition technology has gained a significant attention due its unique attributes along with liveness property yielding very high recognition rates. Vein patterns are segmented into different sub-modalities amongst them most commonly used come from the palm (Zhou & Kumar, 2011), palm dorsal (Joardar, Chatterjee & Rakshit, 2014), wrist (Pascual et al., 2010), or finger (Lee et al., 2010). The sub-dermal nature of veins makes these types of biometrics a highly secure modality (Crisan, 2017). In a vein biometric system, image acquisition is carried out by using near-infrared (NIR) imaging device. The NIR light maps the vein locations, because the hemoglobin in veins absorbs the NIR light. A high contrast image is created by visualization of the vein pattern as shadows appearing over a white background. These high contrast images with vein patterns are used for recognition using various texture feature extraction techniques.

A quick review of the literature elucidates the facts that very high recognition rates are obtained on this biometric trait. Researchers have reported an accuracy of upto 99.4% reinforcing the theoretical claims of high uniqueness of vein patterns (Das et al., 2018). However, the requirement of using sophisticated acquisition devices for obtaining the biometric data makes this modality relatively less popular (Kilian et al., 2020). Moreover, using vein patterns can be a challenge in some cases because of the physiological changes taking place due to ageing and various medical conditions (Oloyede & Hancke, 2016).

### Hand geometry

**Figure 8:**
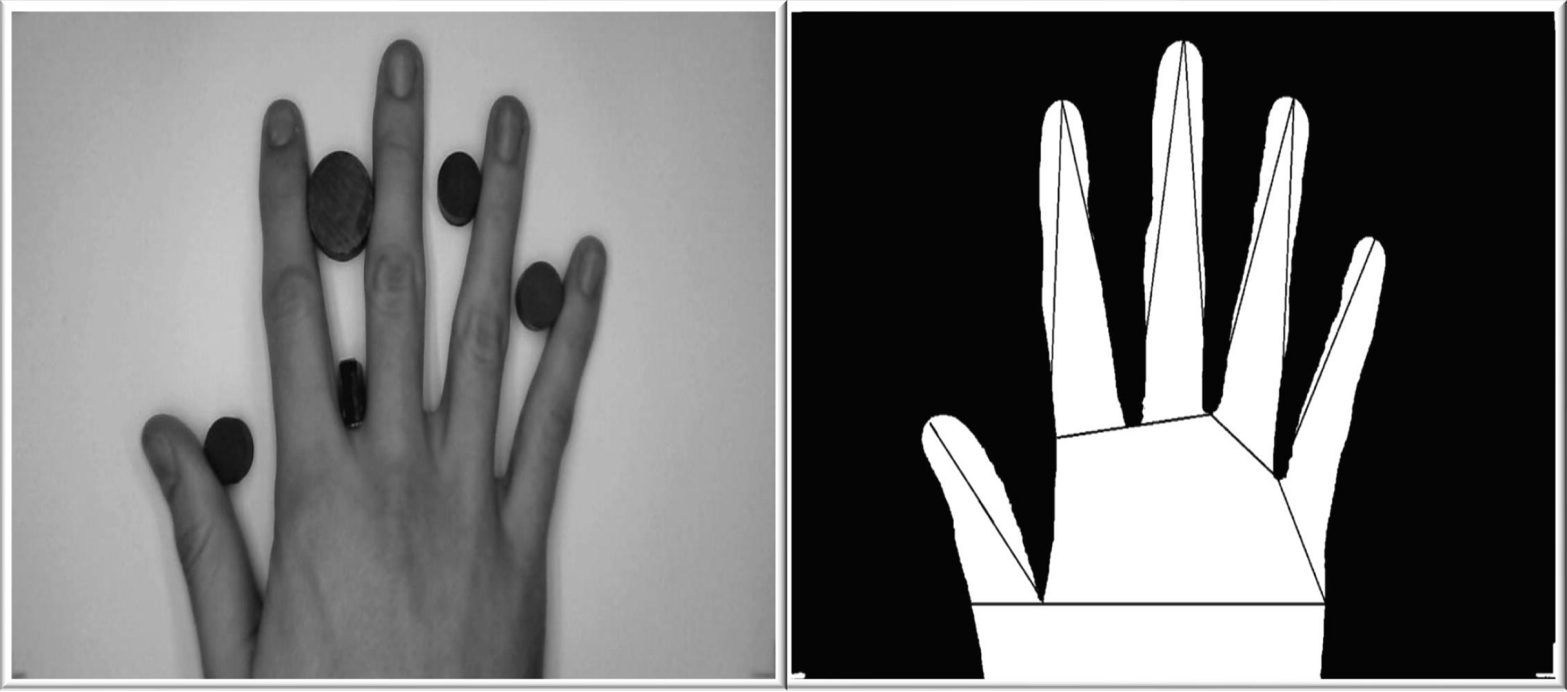
A human hand used for the extraction of hand geometry features.

Hand geometry/shape is a very simple biometric technology that uses the measurements of human hand to verify the identity of the individuals. The measurements include the length, shape and width of fingers and size of palm (Fig. 8). The biometric systems employing hand geometry are widely used as they have a high public acceptance (Babich, 2012; Jain, Dass & Nandakumar, 2004, Sharma et al., 2015). However, it should be noted that the systems based on this technology are not scalable as the hand geometry is not highly unique (Oloyede & Hancke, 2016). Nevertheless, it is widely used at places providing access control, where the main objective is to find out if someone is illegally trying to gain access to someone’s personal identification. A hand reader guarantees that a worker is actually available at a place where he is meant to be. It is also used for implementing time attendance of the employees and helps in stopping the employees from buddy punching (which takes place commonly with fingerprint technology). Hence, the payroll accuracy of a company is guaranteed with a higher probability when hand geometry is used (Babich, 2012).

Due to the lack of ability to differentiate between the people effectively, the usage of hand geometry is somewhat limited and typically used in conjunction with other biometric modalities for improved recognition rates. Some recent contributions on hand geometry show that an EER of upto 0.31% has been achieved by Sharma et al. (2015) with upto 50 distinct users. A novel contactless sensing system (Kanhangad, Kumar & Zhang, 2011) based on multi sampling has been proposed, which has been used to authenticate a database of 100 people representing upto 200 hands with about 50% improvement in the recognition rates (Oloyede & Hancke, 2016). Nevertheless, the technology is not as accurate as its counterparts and thus is not very useful in a standalone setting for large scale deployment for commercial purposes.

### Finger knuckle print/inner knuckle print (FKP)

**Figure 9:**
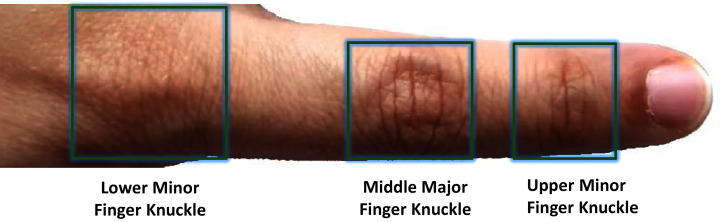
Finger dorsal knuckle print around the joints.

Finger knuckle print is one of the emerging hand based modalities used for biometric verification of the individuals (Kumar & Ravikanth, 2009). The finger knuckle patterns can be easily acquired using contactless devices. In contrast to the more established modalities such as fingerprints requiring high resolution imagery, the knuckle patterns can be easily captured using low-resolution samples (Zhang, Lu & Zhang, 2018). Additionally, the patterns on the outer surface of the knuckle appear at an early stage and survive for a longer periods of time and are specifically useful for the workers, labourers, cultivators, etc., whose fingerprints are more susceptible to damage due to the nature of work (Yang, Yu & Liao, 2009). In a biometric system based on finger knuckle, the physiology which differentiates two different people is due to the lines, creases and texture of the knuckle print that lie at the three knuckle joints of the fingers (Jaswal, Kaul & Nath, 2016) (Fig. 9). These lines appear before birth and rarely change over an individual’s lifetime.

The knuckle print is a biometric that can be acquired without any physical contact with any sensor. Therefore, the chances of spoofing are significantly reduced. These are highly stable for individuals from various age groups; however, their widespread usage is still not reported. A quick survey of the literature shows that researchers have obtained a high accuracy on the identification of persons using knuckle prints with an overall accuracy of upto 98% in real time on a dataset of size 7,900; FAR of 0.062% and FRR of 0%. Given the ability to obtain data for finger knuckles contactlessly, the ease in acquisition process, invariance of patterns to emotions and behavioral aspects, and a wide acceptability socially, there is potential in using this technology on a large scale undoubtedly. However, research is needed on improving the identification results for a widespread deployment.

## Feature extraction techniques for hand multibiometric systems

**Figure 10:**
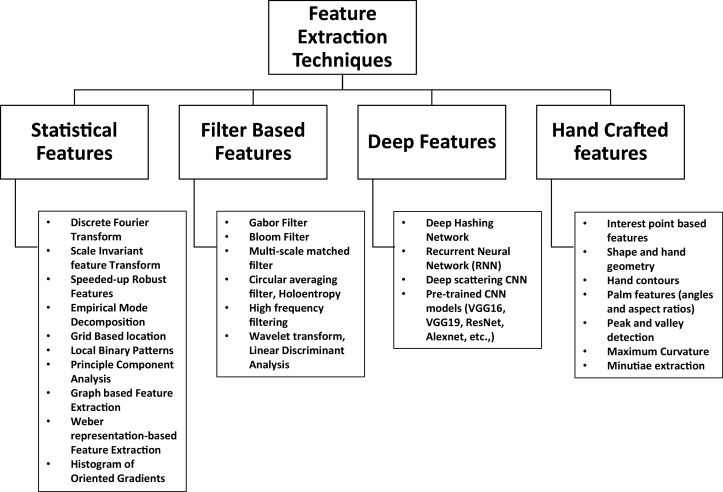
Taxonomy adopted for feature extraction techniques.

A significant volume of literature exists with regards to the design of hand multibiometric systems. It is well known that for any system, the feature extraction methodology is the most fundamental aspect that governs the recognition performance achieved by the systems. In this context, a survey of the literature reveals that all the methods can be mainly divided into the following main categories: 1. Statistical features, 2. Filter based features, 3. Deep features and 4. Hand crafted features, leading to a taxonomy, which is shown in Fig. 10.

### Statistical features

Texture is a spatial property that is generally valid over a larger spatial neighborhood. In order to capture the spatial characteristics and dependence of the images, some statistical measures can be used in order to summarize the patterns indicated in the images. Using statistical methods typically yield good quality image descriptors with the requirement of a smaller memory footprint for maintaining the templates of the registered users. Various authors have worked on proposing statistical methods for feature extraction (Summary presented in Table 3).

**Table 3:** Summary of literature on statistical features.

Publication	Modalities	Feature extraction	Database	Fusion level	Recognition rates
Aoyama, Ito & Aoki (2014)	Multi-instance FKP	2D DFT	7920 images (PolyU FKP database)	Score level	EER-0.321%
Perumal & Ramachandran (2015)	Palm print, FKP	SIFT, SURF, EMD	PolyU dataset (7920 images)	Score level	Acc-99.54%, FAR-0.761%, FRR-0.83%
Veluchamy & Karlmarx (2016)	Finger vein, Finger Knuckle	Grid based location	SDUMLA-HMT (Finger vein), IIT Delhi FKP images	Feature level	Acc-96%
Yang & Sun (2016)	Palm print, Palm vein	Local binary patterns	CASIA dataset (100 persons)	Feature level	EER-0.077%
Srivastava et al. (2016)	Palm veins, Dorsal vein	Palm phalenges, Mean, Absolute deviation	NSIT Palm print Database 1.0	Score level	Acc-98.2%
Chaudhary, Srivastava & Bhardwaj (2016)	Palm print, Dorsal vein	Gaussian function	IIT Delhi Palm print, Bosphorus Hand Vein Database	Feature level	FAR-99.83%
Bhilare et al. (2018)	Palm vein, Finger vein	Local binary patterns	PolyU dataset, VERA Palm print dataset, CASIA dataset	Score level	Acc-100%, EER-0.13%
Vishi & Mavroeidis (2018)	Fingerprint, Finger vein	–	SDUMLA-HMT dataset	Score level	EER-0.0001%
Yang et al. (2018b)	Fingerprint, Finger vein	Cancelable template generation, DFT	FVC2002 (800 images), FVC2004 (800 images)	Feature level	EER-0.12%
Korichi et al. (2018)	Palm print, Palm vein, FKP, Finger vein	Principle component analysis	PolyU dataset (7920), SDUMLA-HMT finger vein dataset (100 persons)	Score level, Feature level	EER-0.007%
Yang et al. (2018a)	Finger vein, Finger dorsal texture	Weber representation,Cross section asymmetrical coding	CASIA (6000 images), PolyU (2515 finger dorsal images), USM(492 fingers)	Feature level	CASIA (EER-3.24%), PolyU (EER-2.72%), USM (EER-3.83%)
Zhang et al. (2019)	Fingerprint, finger vein, finger knuckle	Graph feature extraction	17550 images	Feature level	Acc-99.8%
Veluchamy & Karlmarx (2020)	FKP, Palm print	Entropy weighted gradient decomposition	IIT Delhi FKP and Palm print	Feature level	Acc-95%, FAR-0.1%, FRR-0.1%
Lv et al. (2020)	Fingerprint, Finger vein	Local binary patterns	1500 images	Feature level	EER-0.95%
You & Wang (2019)	Fingerprint, Finger vein	Template fusion and coding	100 images	Feature level	GAR-95%, FAR-0.4%

Aoyama, Ito & Aoki (2014) proposed a FKP recognition algorithm based on block matching using phase correlation where the phase information was extracted from the 2D DFT (Discrete Fourier Transform) of the images. Good recognition results were obtained with a high main-to-side lobe ratio of correlation and an EER of 0.321%. Perumal & Ramachandran (2015) proposed a method to fuse the palm print and FKP of individuals using interest point based techniques including SIFT (Scale Invariant Feature Transform), SURF (Speeded-up Robust Features) and EMD (Emiprical Mode Decomposition) with even better results but it should be noted that EMD is an iterative algorithm and is not very suitable for real-time implementations. Veluchamy & Karlmarx (2016) made use of FKP and finger veins to design a biometric system in which repeated line tracking is performed followed by grid operation, yielding a set of features, which are used for classification using support vector machines (SVM). Although the overall results are good, there is a margin for improvement before the method can be deployed for commercial usage. Yang & Sun (2016) proposed a biometric system making use of palm print and palm veins, in which local binary patterns were used for feature extraction. The method achieves good results; however, a wider validation on a larger dataset is required for a thorough evaluation of the proposed method. Srivastava et al. (2016) performed the fusion of palm-phalages, palm print and dorsal hand vein using some statistical features (average absolute deviation, mean features and Gaussian membership) followed by classification using SVM, KNN (k-nearest neighbors) and random forest. However, the main aim of the authors was to introduce a new dataset for carrying out biometric research.

Chaudhary, Srivastava & Bhardwaj (2016) used palm print and dorsal hand vein for recognition, performing feature extraction using Gaussian membership function. Bhilare et al. (2018) made use of the images from hand vein based modalities by performing an ROI extraction, followed by the use of CS-LBP (Center Symmetric Local Binary Patterns) as texture features. They obtained very good results, concluding in their research that the palm vein and finger vein images yield better results rather than using the vein structure of the entire hand for recognition. Vishi & Mavroeidis (2018) made use of fingerprint and finger vein for performing recognition. The paper was aimed at a combination of score normalization and fusion techniques on the two aforementioned modalities, concluding that the hyperbolic tangent score normalization technique achieves the highest recognition rates. Yang et al. (2018b) aimed at improving the template protection in a multibiometric system in which fingerprint and finger veins were used. They made use of DFT based features, achieving very good recognition rates while ensuring the security of the templates. Korichi et al. (2018) proposed a multibiometric identification system with modalities composed of images obtained from both visible and near-infrared light. Feature extraction is performed using PCA (principal component analysis), with results obtained on various datasets, showing a high precision.

Yang et al. (2018a) proposed a Weber representation based feature extraction method for feature extraction from finger veins and dorsal veins. They performed feature level fusion and validated their results on several datasets, achieving very good results. Zhang et al. (2019) made use of multiple finger based modalities, followed by feature extraction using graph based methods. They achieved very good results, showing a high potential of employing graph theory for designing biometric recognition systems. Also, the results are validated on a large dataset, elucidating on the statistical significance of the achieved performance. Veluchamy & Karlmarx (2020) proposed a multibiometric system based on FKP and palm print in which they used HoG (histogram of oriented gradients) features. These are also part of the MPEG-7 standard and thus are used widely for multimedia application. Good recognition rates have been achieved; however, there is a margin of improvement in the overall recognition rates that could possibly be achieved using more specialized descriptors. Lv et al. (2020) made use of LBP based image descriptor for feature extraction from the fingerprint and finger vein images. Feature level fusion was employed to combine the fingerprint and finger vein patterns into a single image, followed by the use of contrast enhancement techniques, and later performing LBP giving good results. You & Wang (2019) discussed about the classical disadvantage of the fuzzy vault scheme in terms of potential attacks carried out by the attackers. They mitigated this problem by performing the fusion of fingerprint and finger vein templates, followed by the projection of feature points on a rectangular grid. The fuzzy vault scheme is later used for encoding and decoding purposes. Good results are obtained along with experimental evidence of the security of the proposed scheme.

### Filter based features

Several feature extraction methods exist in the literature that are based on first filtering the images with a specific mask (filter) or a set of filters and then estimating the texture of the images based on models or statistics of the filter outputs. Typically, these methods result in a decomposition of the images giving a large amount of data based on the number of filters and their parameters (more descriptive with respect to some texture related characteristics such as edges, scales, angles, etc). Later, the decomposed information is summarized using some statistics, which enhance the description of the images with respect to the filter parameters. The literature is rich in terms of the use of filter based methods for texture feature extraction (Summary in Table 4).

**Table 4:** Summary of literature on filter based methods.

Publication	Modalities	Feature extraction	Database	Fusion level	Recognition Rates
Chin et al. (2014)	Fingerprint, Palm print	Gabor filters	FVC2004 DB1 (800 images), PolyU databse (7750 images)	Feature level	EER-0.001%
Khellat-Kihel et al. (2016)	Finger vein, Fingerprint, FKP	Gabor filters	PolyU database (7920 images)	Feature level, Decision level	EER-0.04%, FAR-99.53%
Gupta, Srivastava & Gupta (2016)	Hand geometry, Vein patterns	Multi-scale matched filter, Variational calculus (vein), Hand crafted features (hand geometry)	IITK-Pdv dataset (538200 images)	Score level	CRR-99.34%, EER-1.87%
Verma & Dubey (2017)	Hand vein, Finger vein, Palm vein	Filtering, holoentropy thresholding	SDUMLA-HMT (finger vein), Bosphorus (hand vein), CASIA (palm print)	Score level	Acc-89.9%
Bharathi & Sudhakar (2019)	Finger vein, palm vein	Gabor filters, gradient based methods	SDUMLA-HMT (Finger vein, palm vein)	Score level	FRR = 0%, Accuracy = 99.5%
Kauba, Prommegger & Uhl (2019)	Finger vein, Hand vein	Gabor filters, Principal curvature, Maximum curvature, SIFT	PLUSVein (Finger vein, hand vein)	Score level	EER-0.03%
Jaswal & Poonia (2020)	Palm print, FKP	Wavelet transform	PolyU FKP (7920 images), CASIA palm print (5502 images)	Score level, Rank level	EER-0.26%, Acc-100%
Li et al. (2021)	Finger vein, FKP	Gabor filters	Data-multi (Finger vein, FKP)	Feature level	EER = 0.63, Acc = 99.6%

Chin et al. (2014) predominantly focus on multibiometirc template protection. They have proposed a three-stage hybrid method. In order to obtain a fused template, fusion of fingerprint and palm print images is done at feature level, followed by applying random tile technique to obtain random features. These random fused features undergo discretisation, hence generating a secure template bit string. Khellat-Kihel et al. (2016) pointed out that the multimodal biometric system improves the accuracy significantly but on the other hand, they tend to have a larger memory footprint and result in longer execution times. Therefore, they proposed the extraction of features using Gabor filters, followed by feature selection using linear discriminant analysis (LDA) giving good recognition results. Gupta, Srivastava & Gupta (2016) proposed a hand geometry and vein pattern based method in which gradient based variational approach is used for the extraction of veins. Matching is performed using global approach in which Fourier Mellin transform is used, thus avoiding the issues such as non-uniform illumination, noise, etc. Hand geometry features are obtained using hand crafted features. The authors have carried out validation of methods on a large dataset with statistically significant results.

Verma & Dubey (2017) proposed a multimodal vein based recognition system in which an ROI (region of interest) is identified followed by vein enhancement using circular averaging filter and holoentropy thresholding. The results reported by Verma & Dubey (2017) are not very good; however, the feature extraction procedure is not precisely mentioned, and thus the quality of features employed cannot be fairly assessed. Bharathi & Sudhakar (2019) made use of hand vein based biometric modalities for performing recognition. Feature extraction was performed using Gabor filter and gradient based methods with matching performed using the Euclidean distance metric. Although good results are obtained, the authors have mentioned that using some other fusion techniques can improve the results. Furthermore, researching on a more relevant distance metric could potentially be useful for matching purposes. Kauba, Prommegger & Uhl (2019) proposed a contactless device to acquire images corresponding to hand-based biometric modalities, which make use of vein patterns for recognition. The authors collected a dataset for evaluation and also used various methods including Gabor filter, high frequency filtering, and interest point based methods for feature extraction. The proposed device achieves good results and exhibits potential for usage for recognition tasks. Jaswal & Poonia (2020) made use of palm print and finger knuckles to design an authentication system. They performed an ROI extraction from the respective images followed by line ordinal pattern based encoding of the images. Later, feature extraction is performed using criterion wavelet transform and feature selection is performed using linear discriminant analysis and search based methods yielding very good recognition rates. Li et al. (2021) proposed a joint discriminative feature learning framework in which the directional features are estimated using Gabor filters, which are later fed into an optimization framework for feature learning that maximizes the inter-class variation and minimizes the intra-class variation among samples. Finally, block-wise histograms of learned feature maps are used for recognition purposes, giving very good overall recognition accuracy of about 99.65%.

### Deep features

Over the last couple of years, the Deep Convolutional Neural Networks have dominated significantly in terms of the extraction of features and for performing the classification tasks or solving recognition problems. This is because of their robust framework, having an incredible ability to learn from the training data and adapt the designed networks to solve complex problems. Even in biometric systems, the employment of deep learning has seen a significant surge and is producing very good results in comparison to the other methods that have been used previously (Summary in Table 5).

**Table 5:** Summary of literature on filter based methods.

Publication	Modalities	Feature extraction	Database	Fusion level	Recognition rates
Zhong et al. (2018)	Palm print, Dorsal hand vein	Deep hashing network (Palm print), Hand crafted features (Vein)	PolyU dataset, GPDS vein dataset	Feature level	FAR-0.0495%
Zhong, Shao & Du (2019)	Palm print, dorsal hand vein	Deep Hashing Network	NCUT database, GPDS database	Sensor level, Feature level, Matching score, Decision level	FAR ≈ 0%, FRR ≈ 0%
Toygar, Babalola & Bitrim, 2020	Palm, dorsal and wrist vein	Hand crafted, CNN based	FYO Vein database	Feature level	Acc-100%
Chen & Wang (2018)	Hand shape, Palm print	Block statistics	VIP-CC database (hand and palm images)	Decision level	FAR-0.0095%, FRR-5.7692%, EER-7%
Mehdi Cherrat, Alaoui & Bouzahir, 2020	Fingerprint, Finger vein	CNN	SDUMLA-HMT (41,340 images)	Feature level	Acc-99.59%
Chen et al. (2019)	Palm vein, Palm print	CNN	540 images	Feature level	Acc-99.97%
Choudhury, Kumar & Laskar, 2021	Dorsal hand	Alexnet, Resnet	890 images	Rank level	Av Error-0.0097

Zhong et al. (2018) proposed the use of DHN (deep hashing network) for palm print encoding into 128-bit codes, and BGM (biometric graph matching) to encode dorsal hand vein images into three discriminant features. Later, feature level fusion was used with very good recognition rates, with EER of upto 0%. Toygar, Babalola & Bitrim (2020) proposed a deep architecture with five hidden layers, each comprising convolutional, batch normalization and pooling layers to design a multibiometric system based on palm, dorsal, and wrist veins. The results when compared with several other methods including hand crafted features and Gabor filters, show very good results, elucidating on the potential of using deep learning methods for multimodal biometrics. Zhong, Shao & Du (2019) proposed a deep end-to-end trainable hashing network that takes an image at the input and outputs a binary code corresponding to the respective image. Matching can be performed by comparing the binary codes corresponding to the training images with the image given as input to the network. The method achieves very good results, showing promise in employing the neural networks based techniques for encoding the images. Chen et al. (2019) introduced a low cost personal identification system consisting of near infrared and visible LED (light emitting diodes). An adaptive feedback control was used to control the brightness of the diodes. The images acquired were preprocessed, with feature extraction performed using a deep scattering CNN, giving good recognition rates. Mehdi Cherrat, Alaoui & Bouzahir (2020) proposed a system for recognition using the CNN models in a multibiometric setting with a fusion of finger vein and fingerprint. Good recognition rates were obtained using the proposed strategy, with a conclusion that the use of preprocessing improves the recognition rates. Choudhury, Kumar & Laskar (2021) made use of index, middle, and ring fingernail plates for extracting biometric features from the images using three customized pretrained models: Alexnet, Resnet and Densenet. An adaptive fusion technique based on score and decision level is used for the purpose of fusion of features from dorsal hand followed by exhaustive experiments to assess the efficacy of the proposed technique, giving very good recognition results with a minimum average rate of 0.0097%.

### Hand crafted features

Handcrafted features are typically those which are used with more traditional machine learning algorithms for performing classification tasks. More commonly, these features can be easily correlated to statistical features. In this specific article, we define the handcrafted features as those features that are obtained as a combination of statistical and physical properties of the images such as hand size, finger size, etc. Such combinations are typically obtained when at least one of the biometric traits used for recognition is hand geometry. Although the literature on such techniques is limited, a summary of relevant contributions made using such methods is presented in Table 6.

**Table 6:** Summary of literature on feature extraction using hand crafted features.

Publication	Modalities	Feature extraction	Database	Fusion level	Recognition Rates
Sharma et al. (2015)	Hand shape, Hand geometry	Hand crafted features	JUET contact database (50 subjects), IITD contact less dataset (240 subjects)	Score level	EER-0.31%
Anitha & Rao (2016)	FIKP, Hand geometry	Hand crafted features	PolyU dataset (7920 images)	Feature level	ERR-0.8%
Jaswal et al. (2019)	FKP, Palm print, Hand print	Hand crafted feature, shape features	CASIA, IIT Delhi, PolyU	Feature level	EER-0.01%, CRR-100%
Gupta & Gupta (2018)	Fingerprint, Dorsal vein, Hand geometry	Hand crafted	2000 hand slap images, 2000 IR hand images	Matching score	EER-0.72%, CRR-100%
Khodadoust et al. (2021)	Fingerprint, Finger vein and FKP	Maximum Curvature	924 Finger prints, 924 Finger veins and 924 FKP	Matching level, Score level	–

Sharma et al. (2015) performed identity verification using the shape and geometry of hands using the contour of the hands. The hand contour is initially aligned followed by the calculation of peaks and valleys, and the extraction of the finger feature points by calculating the Euclidean distance between the reference point and all the feature points. The proposed method shows promise with good recognition rates achieved over two datasets. However, their sizes are small and the methodology requires extensive validation over larger datasets for a statistically significant conclusion. Anitha & Rao (2016) made use of FKP and hand geometry to propose a multibiometric system. They performed ROI extraction from the pictures followed by the use of LBP as texture features for the finger knuckles and hand geometry features using hand crafted features. The process for the extraction of these features includes the identification of six points on the hand to extract the angle features and the aspect ratio of the palm, followed by the calculation of Euclidean distance between the template and the acquired image for matching. Experiments show that the best performance is achieved using a feature level fusion of the FKP and hand geometry features. Jaswal et al. (2019) laid down the idea of using multiple biometric traits for recognition using a single sensor. A device having the ability to capture the FKP and palm print was used for acquiring the image. Processing of the images was done by the extraction of an ROI followed by a transformation using texture code matrix. Hand registration was done by detecting the feature points (peaks and valleys) and also the detection of knuckle point followed by deep multiscale matching giving very good recognition rates. Gupta & Gupta (2018) proposed a system that captures slap fingerprints and hand dorsal image at the same time. Slap segmentation is performed by making use of the finger location and hand type. Matching of scores are generated by matching the slap fingerprints, palm dorsal vein and hand geometry that are fused for the purpose of authentication yielding good recognition results. Khodadoust et al. (2021) worked on the fusion of fingerprint, finger veins, and FKP using an experimental setup, which obtained the 3D reconstruction of the above-mentioned traits followed by maximum curvature based feature extraction. They obtained good overall identification results, with validation carried out on 66 users showing promise for the use of their method for authentication purposes. The most significant claimed advantage of the proposed method relates to the experimental protocol as the method relies on a contactless and hygienic way of acquiring multibiometric traits.

Now that we have analyzed the feature extraction techniques, which have been presented in the literature, it is important to note that there are a several points to compromise a biometric system. It is very important for a biometric system to be unsusceptible to attacks and loss of template by adversaries. To deal with the issues related to the security of a multibiometric template, we now analyze the existing work on multibiometric template protection.

## Multibiometric template security

A multibiometric system uses multiple biometric traits (*e.g*., fingerprint, face, and finger vein) to recognize a person (Ross, Nandakumar & Jain, 2008), hence improving the reliability and accuracy of biometric systems. However, adequate attention has not been paid towards making the multibiometric templates secure. There are several ways to compromise a biometric system (Ratha, Connell & Bolle, 2001) and loss of a biometric template information to unauthorized individuals possesses security and privacy threats (Nagar, Nandakumar & Jain, 2011; Mirza et al., 2014) due to following reasons:

- *Intrusion attack at biometric system:* If an adversary gets an unauthorised access into a biometric system, he can easily access the stored biometric template of a user. This information can be used to get an illegal entrance into the biometric system in which the user is enrolled by either reverse engineering the template and disguising as this user or replaying the stolen template.
- *Database Linkage*: Once an adversary gets hold of a template, it can be easily determined if the two templates from different databases belong to the same person or not. Moreover, different databases hold separate parts of data regarding that person. Consequently leading to more data theft and more difficult identity-related attacks.

**Figure 11:**
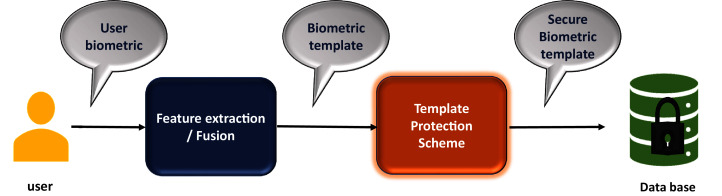
Biometric authentication system incorporating template security.

**Figure 12:**
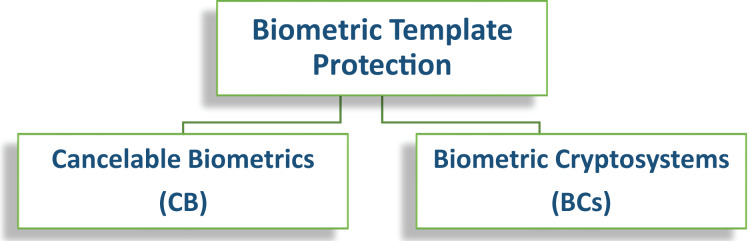
General categorization of template protections schemes.

Keeping this in view, the security of a multibiometric system is very critical as it contains information regarding multiple biometric traits of the same user and it should be shielded from an unauthorized access (Chin et al., 2014; Rathgeb & Busch, 2012). Therefore, there is a need for a secure template that must be irreversible and unlinkable (Davida, Frankel & Matt, 1998; Ratha et al., 2007; Bolle, Connell & Ratha, 2002; Juels & Sudan, 2006; Sutcu, Li & Memon, 2007a, Sarkar & Singh, 2020; Bharathi & Mohana, 2019) (Fig. 11). Biometric template protection schemes can be categorized into two main classes (Fig. 12) (Rathgeb & Uhl, 2011; Sandhya & Prasad, 2017): 1. Cancelable Biometrics (CB), 2. Biometric Cryptosystems (BCs). These schemes offer various advantages over a generic biometric systems. A few most important advantages are summarized in Table 7.

**Table 7:** Advantages of template protection.

Advantage	Description
Secure template/Privacy protection	Reconstruction is hardly feasible in biometric cryptosystems and cancelable biometrics as the original biometric template is obscured.
Secure key release	Key release mechanisms provided in cryptosystems are based on biometrics.
Pseudonymous authentication	The encrypted identifier that is used for authentication is also a pseudonymous identifier.
Revocability of templates	Multiple instances of templates can be generated from the same biometric data.
Enhanced security	Traditional attacks are mitigated with the usage of cancelable biometrics and biometric cryptosystems.
Social acceptance	The social acceptance of biometric applications is expected to increase with the use of cancelable biometrics and biometric cryptosystems.

### Cancelable biometrics

Cancelable biometrics (CB) refer to distortion of biometric features that are intentional and systematically repeatable in nature to protect sensitive user-specific data (Ratha, Connell & Bolle, 2001). Cancelable biometric transforms are those that are used to transform the original biometric samples such that the resulting data is computationally hard to recover. When the user registers in the system, his biometric sample is transformed using a one-way transformation and saved in the database. This transformation is chosen from an identification word that is specific to the user. In the verification step, the query template is used to generate a transformed template that is compared with the saved template in the database followed by the verification process. The literature on unimodal cancelable biometric systems is very rich but there are inherent problems with such systems including intraclass variability, variation in data quality and a significant similarity in interclass samples. In contrast to such systems, the multimodal biometric systems combine the features of various biometric traits to generate the templates, which are more secure and thus, resistant to various threats and attacks. The main advantages offered by the multibiometric systems are greater security, accuracy, noise sensitivity, and resistance to spoof attacks. Table 8 presents a brief summary of the work done in the domain of cancelable multibiometircs.

**Table 8:** Related work on cancelable multibiometric systems.

Year	Authors	Description
2012	Paul & Gavrilova (2012)	Multibiometric template protection using PCA as a transform based tool
2013	Paul & Gavrilova (2013)	Multibiometric template protection using Gram-Schmidt transformation
2014	Chin et al. (2014)	Hybrid template protection using feature fusion and random tiling transformation
2014	Paul & Gavrilova (2014)	Multibiometric template protection using Gram-Schmidt transform, PCA and rank level fusion
2017	Gomez-Barrero et al. (2017)	Homomorphic probabilistic encryption for cancelable biometric template generation
2018	Yang et al. (2018b)	Fusion based cancelable multibiometric system
2018	Kaur & Khanna (2018)	Random distance method for obtaining secure biometric templates
2018	Gomez-Barrero et al. (2018)	Bloom filter based cancelable biometric features
2019	Dwivedi & Dey (2019)	Cancelable features followed by score level fusion
2020	Walia et al. (2020)	Cancelable deep feature, followed by adaptive graph fusion
2020	Chang et al. (2020)	Novel bitwise encryption scheme to generate biometric template

Researchers have made several contributions on multibiometric template protection employing cancelable biometrics. Paul & Gavrilova (2012) proposed a method in which two-fold random selections are made from each biometric trait, followed by a feature level fusion. Random projection of each fold is obtained followed by PCA (principal component analysis) and later K-means clustering to generate the single template for individual biometrics. Later, LDA (linear discriminant analysis) is applied to further improve discriminability of the features. Final authentication is carried out using a classifier. Another variant of the technique proposed in Paul & Gavrilova (2013) makes use of Gram–Schmidt transformation instead of PCA, along with some other minor modifications in the pipeline. The authors have validated the cancelable property of the proposed method, while giving good authentication results in a multibiometric setting. Furthermore, the authors improved the results by proposing a methodology in which both Gram–Schmidt transformation and PCA were used followed by a rank level fusion for performing final authentication of the users (Paul & Gavrilova, 2014). Chin et al. (2014) proposed a three-stage hybird template protection scheme. They have performed the fusion of palm print and fingerprint on the feature level, followed by the use of random tiling technique to extract unique features. Finally, the fused random features undergo 2^*N*^ discretisation to produce the template bit string. The approach addresses the criterion for template protection with an improved EER as compared to unimodal biometric sytems, though it is slightly higher with reference to multimodal systems. Gomez-Barrero et al. (2017) made use of homomorphic probabilistic encryption to generate the biometric templates along with fusion at three different levels. A complexity analysis was also carried out to assess the feasibility of the proposed method for real-time implementation. Moreover, feature level fusion is also employed yielding more secure and better cancelable biometric features. Kaur & Khanna (2018) proposed a template transformation method named random distance method that yields privacy preserving, revocable and discriminative pseudo biometric identities, with about 50% reduced memory footprints. Yang et al. (2018b) proposed a multibiometric system in which the fingerprint based minutae features and finger vein features are extracted followed by their respective binary features, and then performing feature level fusion in three different ways. The method obtained secure biometric templates with good recognition results. Gomez-Barrero et al. (2018) showed the use of Bloom filter based protection schemes while elucidating that it is not a straightforward task. A statistical analysis of unprotected templates is carried out to estimate the main parameters of such schemes. Dwivedi & Dey (2019) proposed a method to obtain cancelable templates by using log-Gabor filters with phase quantization, followed by the generation of biometric codes. Score level fusion from multiple biometric templates are used for authentication yielding better results in comparison to unibiometric systems with better accuracy. Walia et al. (2020) proposed a method to obtain cancelable features using deep neural networks that are fused using adaptive graph based fusion method. The proposed method is used to obtain multimodal unified templates, which are empirically demonstrated to be robust to adversary attacks. Chang et al. (2020) proposed an authentication approach in which bit-wise encryption scheme is used to transform a biometric template into a secure template using a secret key, which is generated from another template. The scheme fully preserves the number of bit errors in the protected and original template, ensuring that the recognition performance is the same as that in the case of unprotected templates.

### Biometric cryptosystems

Biometric cryptosystems (BCs) refer to designs that securely generate digital key from a biometric or bind a digital key to a biometric (Cavoukian & Stoianov, 2009). To overcome the shortcomings of traditional verification methods, which were based on password-based key-release, BCs bring about a considerable security benefit by offering biometric-dependant key-release since the biometrics have a strong link with the user’s identity (Uludag et al., 2004; Jain, Ross & Uludag, 2005; Rathgeb & Uhl, 2011). At the same time, combining biometrics with cryptography and extracting the keys is not that straightforward due to variations present in a biometric data. Most of the BCs require helper data that contains additional information about the biometric and is used to generate or retrieve a key (Jain, Nandakumar & Nagar, 2008; Ali & Khan, 2014). A helper data must not reveal significant information about original biometric templates. Table 9 presents a brief summary of the biometric cryptosystems.

**Table 9:** Related work on biometric cryptosystems.

Year	Authors	Description
2007	Sutcu, Li & Memom (2007b)	Protection of face and fingerprint templates
2008	Nandakumar & Jain (2008)	Multibiometric template security using fuzzy vault
2008	Camlikaya, Kholmatov & Yanikoglu, 2008	Encoding of fingerprint with voice features
2009	Fu et al. (2009)	Multibiometric fusion at cryptographic level
2011	Nagar et al. (2011)	Feature level fusion for fuzzy vault and fuzzy commitments
2015	Li et al. (2015)	Biometric cryptosystem using computational security and information security, with decision level fusion
2016	Kumar & Kumar (2015)	Multibiometric system based on cell array for storing has code and keys separately
2019	You & Wang (2019)	Novel fuzzy vault scheme based on the feature level fusion of the fingerprint and finger vein.
2020	Chang et al. (2021)	Multibiometric cryptosystem based on Fuzzy vault and fuzzy commitment
2021	Evangelin & Fred (2021)	Cryptographic model based biometric template protection
2021	Asthana, Walia & Gupta, 2021	Cryptographic key binding for template protection

Sutcu, Li & Memom (2007b) proposed the use of multibiometric features, followed by a *secure sketch* block, making it hard to extricate the original samples from the encrypted features. Nandakumar & Jain (2008) proposed a method to enable template protection using fuzzy vault framework. The authors claim to improve the recognition performance of the system along with enhanced security. Camlikaya, Kholmatov & Yanikoglu (2008) proposed a technique for the fusion of fingerprint template along with behavioral biometrics (voice samples). The algorithm enhanced the security of the biometric system by encoding the fingerprint features within the voice feature vector. The use of voice was motivated by using the property of spoken words used as a password to achieve the desirable cancelable property. Multiple biometric cryptosystems were proposed by Fu et al. (2009) out of which three were used for performing biometric fusion at the cryptographic level. The authors presented no experimental results; however, a detailed theoretical analysis of algorithms, comparison, and discussion were carried out. Nagar, Nandakumar & Jain (2011) provided a feature-level fusion method for both fuzzy vault and fuzzy commitment schemes that simultaneously secure the multiple templates of a user using a single secure sketch. Feature level fusion using multiple characteristics of a user proves to be significant in providing high privacy as compared to the single characteristic biometric systems, since only the fused feature vector is stored on the server database. Further, it requires less storage since only the combined feature vector is stored in the database server. However, it requires additional feature extraction and transformation tools for the heterogeneous features (variable formats based on distance, similarity, etc.). Another hybrid methodology to secure the biometric systems was proposed by Li et al. (2015) in which a combination of computational security and information security principles was implemented. Decision level fusion was done in the proposed cryptosystem for performing recognition. Kumar & Kumar (2015) proposed a multibiometric system based on cell array. Encoding and hash code computation was done using Bose Chaudhuri Hocquenghem (BCH) on the biometric modalities. The data is scattered across the two cells such that the first cell stores the hash code and the second cell stores the key. Moreover, fusion was performed at both decision and feature levels out of which the former shows better results in a multibiometric cryptosystem setting. You & Wang (2019) proposed a novel fuzzy vault scheme, which effectively protects the multibiometric template against location attack, brute force attack, and correlation attack. They performed fusion of fingerprint and finger vein templates. Feature point fusion encoding is done through grid projection, and fusion encodings are applied to construct the fuzzy vault. Chang et al. (2021) proposed BIOFUSE in which fuzzy commitment and fuzzy vault are combined using an encryption scheme. The system makes it difficult for an attacker to gain unauthorized access to the system without doing an impersonation of all the biometric traits at the same instant. The experiments have shown very good recognition rates with a high security. Evangelin & Fred (2021) used a visual shadow creation process to create multiple shadows of one image followed by encoding and decoding using elliptic curve cryptography. Although a very secure model is obtained, the implementation time of the model was significantly expanded. Asthana, Walia & Gupta (2021) made use of a key binding mechanism to generate a secret key using the biometric data of the user, leading to the proposition of a biocrypto system. Novel objective functions are proposed to create the helper data. The local minima of the objective function are taken as anchor points to retrieve the secret key and perform recognition leading to about 98% success rate in recognition even in the presence of limited noise in biometric data.

## Open challenges and future directions

### Challenges

The limitations faced by various researchers in the implementation of biometric systems are listed as under:

- **Aging/Alteration:** It is well known that even when the biometric traits do not suffer any natural changes over a period of time, they are subject to changes due to trauma or physical damage due to cuts, different skin conditions, or other unforeseen events. Even when there is no medical/physical condition, which is liable to cause any damage to the biometrics, the traits change over a period of time (Lanitis, 2010; Trokielewicz, Czajka & Maciejewicz, 2018). This is a challenging problem and as such there is no remedial solution available for such problems other than making use of the biometric traits that are less sensitive to such alterations. Among the hand biometric systems, there are several modalities which exhibit the information not from the skin but the sub-skin structures (veins) that are captured using the IR camera. A mild or superficial skin condition does not affect the vasculature, hence the vein based multibiometric systems have the ability to cater for most of the problems occurring due to trauma on the skin such as cuts. Aging and some other chronic conditions such as hypertension, diabetes, etc., affect the vein biometrics (changing in the diameter of the veins) and thus can impact on the recognition performance of the biometric systems (Xie et al., 2017). However, since these changes do not take place overnight, there are ways to mitigate these problems.
- **Operational problems:** Operational problems refer to the various problems that have the ability to affect the performance of multibiometric systems. These problems can result from various factors such as environment and the methodology of acquisition of the data. The good thing is that most of these operational problems can be mitigated by the acquisition of data multiple times, until a good sample is captured from the acquisition device. For example, if there is excessive moisture in the fingers, the sample captured from the device may be having specular reflection (Auksorius & Boccara, 2017). Such problems can be mitigated if the fingers/hands are cleaned for any moisture before application to the sensor. Apart from environmental conditions, even when the proper methodologies for acquisition of data have been followed, it is possible to face some issues such as alignment. If the biometrics are not properly aligned according to the templates and are, for example, captured at different angles than the templates, then it is possible to handle such problems with one of the modules, such as feature extraction. Therefore, it should be noted that the features should be extracted such that they are invariant to some underlying imaging conditions such as illumination and rotation at the very least.
- **User errors:** The orientation and shifting of the fingers during registration and authentication process significantly affects the performance of the biometric systems based on fingers (Liu et al., 2014; Peng et al., 2012; Dong et al., 2014). Furthermore, during the imaging process, any movement of the fingers or hands causes irregular illumination (Song et al., 2011). As a result, different segments of the fingers/hands get different amount of light absorption and as a result, the quality of the acquired images is not adequate for performing recognition.
- **Biometric finger features:** Another important factor which plays its role is finger features. Studies have shown that in addition to the varying thickness of fingers, certain factors related to finger skin affect the image capturing process, such as skin pigmentation, thickness, hair, etc. (Gupta & Gupta, 2015). Furthermore, studies have shown that the varying thickness of finger skin results in an unequal distribution of light passing through the skin, hampering in the collection of high quality vein patterns.
- **Complexity of fusion:** It is clear that the use of multibiometrics leads to a better recognition and can help in increasing the security of the systems. However, with the use of more than one trait, there are four possibilities of performing the process of fusion on the biometric templates. This leads to critical decision making in any system as it could define the performance as well as the security characteristics of the systems (Dinca & Hancke, 2017; Zhong, Du & Zhong, 2019). Moreover, biometrics is a domain that requires real-time performance and fusion will typically require the system to process a large amount of data in comparison to a unibiometric system (which does not require fusion). The recognition rates by adopting various methods of fusion could vary based on the platforms (*e.g*., mobile, wearable devices, etc.) and architectures (biometric traits, feature extraction methods). Therefore, an exploratory study of the performance yielded by different levels of fusion using standardized platforms and architectures to analyze the best fusion methods is a significant challenge.
- **Security of multibiometric templates:** The security aspect in biometric systems has recently gained a significant traction. There are several techniques that have been explored well including different fusion schemes, convolutional methods using different types of filters, methods for generating secure sketches and fuzzy vault constructs, encryption schemes, etc. In this context, the literature is very rich in regard to the unibiometric systems. The literature on the security of multibiometric systems is very limited and thus there is a lot of scope of work available in this area. The most widely explored methods used for multibiometric systems are derived from transformation based methods and fusion techniques. Generally, the fusion techniques are used in conjunction with a variety of other methods used for inducing security in multibiometric systems. This is because, there are different types of fusion that take place at different stages in the pipeline of multibiometric systems. However, it should be noted that the feature based fusion is the most common type of fusion technique, which is used for enhancing security, as it gives a richer set of features, generally yielding better evaluation metrics for multibiometric systems. It would not be wrong to say that most of the proposed techniques are focused on the use of hand crafted features, along with a mixture of fusion techniques, with a limited focus on representation learning based algorithms.
- **Lack of standard performance measures:** There are several measures that have been used to assess the performance of a biometric system. Due to the lack of a single measure to quantify the system, it is very hard to make a comparison of different methods that have been published in the literature (Ryu et al., 2021; Kumar, Prasad & Raju, 2020; Manisha & Kumar, 2020). There is a need to work on a unified measure that is widely adopted by the researchers to evaluate different authentication systems, and making the literature standardized and valid for performing direct comparisons.

### Future Directions for Improving Biometric Systems

The main future directions of work for the implementation of biometric systems are summarized as follows:

- **Multi-sample registration:** One method that is typically used to solve the problem of variations in the data acquisition for a single person is doing a multi-sample registration, in which multiple samples are captured for a specific trait. In this way, the variations of a single sample are captured and the machine learning methods are appropriately trained to capture this intra-sample variation.
- **Rotation and illumination invariant descriptors:** As discussed previously, one problem that is faced during the sample acquisition is capturing the samples at varying angles. It is practically not possible to always capture the data of the hand biometrics exactly aligned according to the available templates. This could be due to both the acquisition of the template or the sample. An interesting way to mitigate this problem is to work on image descriptors, which are invariant to the image capturing conditions (Riaz et al., 2013). Specifically, if the descriptors are rotation and illumination invariant, this problem can be effectively addressed. However, it should be noted that if the descriptors are illumination invariant, it would not be possible to integrate the information about soft biometrics (such as color of hand) within the template and the respective features.
- **Incremental machine learning (IML):** A very interesting area that has recently gained traction is IML. As discussed, the biometrics are bound to changes over a period of time. Some biometric traits undergo more transformations as compared to the other. If somehow these changes are properly recorded, they can be effectively handled while still yielding higher recognition rates. There are two main methods to solve these issues (Mehrotra et al., 2016): one way is to keep updating the templates as the user is authenticated. Another way is to make the ML algorithm learn new parameters while a new sample is presented for a user. Both these methods are effectively used for incorporating adaptivity to some extent.
- **Fusion based adaptation:** The process of adaptation in biometrics is not only limtied to templates, but it can also be extended to the fusion level. For instance, when performing fusion of multibiometrics, it is possible to perform fusion on the decision level by giving more weightage to the traits which are more stable over a period of time as compared to those which are not. In this context, there are several examples in the literature with regard to adaptive score weighting (Assaad & Serpen, 2015; Sim et al., 2014), score normalization (Khalifa, Gazzah & BenAmara, 2013), adaptive feature weighting (Huang et al., 2015; Xu & Lu, 2015), etc.
- **Soft biometrics:** This paper mainly discusses the literature on hard biometrics in which the physiological biometric traits are used to authenticate the users based on their mathematical modeling. Lately; however, soft biometrics have been gaining attraction since they have the ability to complement the biometric systems with their decision making. The idea behind this concept is that the biometric systems make decision about a specific user based on some characteristics such has skin colour, eye color, height, weight, beard, etc. Interestingly, hand based biometric systems take this liberty to use these soft biometrics to perform recognition. This is because, there are at least two very important characteristics that can be used for recognition in hand based multibiometric systems, *i.e*., skin color and hair. The skin color can be used as a property of the individuals, whereas the presence of hair on the hands or the texture of hands from dorsal view can indicate the gender of the individual. Also, previous studies have shown that the hand measurements, hand length, hand breadth, palm length, palm breadth, etc., can be correlated to the gender of an individual (Rastogi, Murali & Rastogi, 2014). Soft biometrics can be used for recognition in a mixed authentication setting where these are used in conjunction with the hard biometrics for authentication.
- **Multibiometric template protection schemes:** Although the work on multibiometric template protection is limited, this is bound to change in the future given that there is an increasing interest of researchers in extending such algorithms with their variants that yield better results. Moreover, the focus of researchers currently is on using the deep features/algorithms in various domains rather than focusing on the classical classification algorithms (Khan et al., 2019). A notable recent contribution related to cancelable biometrics using deep features is the technique proposed in (Walia et al., 2020), where the authors have used a modified of Resnet model generating deep representation of biometric traits, followed by graph based fusion for generating a unified template. Given that cancelable biometrics make the biometric templates non-recoverable offering a high security, but the recognition rate is expected to be compromised. The method offers better results in comparison to some other methods considered in this paper; however, there is a scope for improvement as can be seen by the performance where the EER of the proposed method is 4.34%. Another potential direction for future research is the use of hybrid methods for template protection, which offers combined benefits of several methods. It should be noted that this requires caution due to the fact that the performance of authentication should still stay real-time and the employment of multiple computationally complex algorithms may require powerful computing resources.

## Conclusions

In this article, we have performed a detailed survey of hand-based multibiometric systems. In this context, various hand-based biometric modalities are discussed, along with a through discussion about various fusion techniques employed and a brief survey of recent work that is being done on template protection schemes in biometric systems. A summary of the main conclusions is as follows:

- The acquisition of biometric templates is a process, which is controlled by the user and thus has the ability to incur some unexpected variations. This can be handled using invariant image descriptors; however, incremental ML is one area that can be explored to solve such problems.
- Lately, hard biometrics can be combined with soft biometrics for authentication purposes. The hand-based modalities give this liberty to extract soft biometrics that can lead to the improvement in biometric security and authentication.
- Most of the work done on the security of biometric templates is employed on unibiometric systems. Multibiometrics in the context of security is largely unexplored with significant margin of improvement for future contributions to the domain.
- Recently, ML algorithms are being used outside their conventional usage as classification tools and multibiometric security is one such area. The potential of deep learning is demonstrated in the literature, with a great margin for improvement.

## Additional Information and Declarations

### Data Availability

The following information was supplied regarding data availability: There is no raw data/code in this paper as it is a survey paper.
